# Understanding Health Information Systems Utilization Across Public Health Centers in Indonesia: Cross-Sectional Study

**DOI:** 10.2196/68613

**Published:** 2025-09-03

**Authors:** Dewi Nur Aisyah, Agus Heri Setiawan, Chyntia Aryanti Mayadewi, Alfiano Fawwaz Lokopessy, Zisis Kozlakidis, Logan Manikam

**Affiliations:** 1 Department of Epidemiology and Public Health, Institue of Epidemiology and Health Care, University College London, 1-19 Torrington Place, London, United Kingdom, 44 207679200; 2 Digital Transformation Office, Ministry of Health Republic of Indonesia, Jakarta, Indonesia; 3 Department of Public Health, Monash University Indonesia, Tangerang, Indonesia; 4 International Agency for Research on Cancer World Health Organization, Lyon, France; 5 Aceso Global Health Consultants Pte Limited, Singapore, Singapore

**Keywords:** public health centers, Puskesmas, health information system, infrastructure, primary health care

## Abstract

**Background:**

The primary health care service in Indonesia consists of 10,260 public health centers (Puskesmas), which play a major role in providing health care in the community, recording and reporting health data using digital health information systems (HIS) or manual reports. The utilization of HIS across Puskesmas is crucial to capture the dynamic evolution of health problems and monitor interventions, thus providing effective primary health care services for the community.

**Objective:**

This paper provides a national-level baseline mapping of HIS utilization in Indonesian Puskesmas. It evaluates the number of HIS used, associated challenges, and contextual factors influencing system adoption.

**Methods:**

A cross-sectional survey was carried out covering all Puskesmas across 34 Indonesian provinces between January and February 2022. The questionnaire covered a list of HIS used by Puskesmas, which developed the HIS, and the utilization and challenges during HIS implementation. Descriptive statistical analysis and bivariate analysis were applied.

**Results:**

A total of 2606 (25.5%) public health centers across 34 provinces participated in this study. On average, Puskesmas reported using 30 different HIS platforms, with notable variation across provinces and islands. Most systems (n=62,060, 72.94%) were developed by national ministries, though local governments and third parties also contributed. Despite 91.5% of respondents reporting that HIS aligned with their needs and 90% claiming data use for decision-making, many centers faced operational barriers: 49% (n=132,300) of systems required excessive data entry, 33% (n=89,100) experienced frequent downtime, and 29% (n=78,300) lacked automated analysis features. In terms of the infrastructure supporting HIS implementation, 9.45% (n=138) of Puskesmas have no access to the internet, while only 28.9% (n=422) have access to robust and efficient internet connections. As for the human resources, the study reveals that each health personnel manages up to six different HIS for data reporting tasks, 74.30% (n=1133) of Puskesmas only received training at the initial system’s implementation stage, and 80.51% (n=1225) of respondents report the existence of an informal knowledge transfer process among the staff. The bivariate analysis shows that Puskesmas with the characteristics of being located in Java island and urban areas possessed higher accreditation levels, had more training and knowledge transfer, and had a greater chance to use >30 HIS.

**Conclusions:**

This descriptive study highlights substantial fragmentation in Indonesia’s HIS environment and reveals critical disparities in system infrastructure, usability, and workforce capacity. Recommendations should be tailored to different contexts: offline-compatible systems and basic digital literacy training are needed in rural areas, while urban Puskesmas may benefit from advanced integration and analytics tools. Future research should address HIS interoperability, impact assessment, cost-effectiveness, and qualitative user experience through longitudinal and mixed methods studies to guide Indonesia’s digital health transformation.

## Introduction

Health information systems (HIS) are defined as a set of structures—including data, information, indicators, procedures, devices, technology, and human resources—that are interrelated and managed in an integrated manner; they provide information support for the decision-making process, health program planning, implementation monitoring, and evaluation at every level of health administration [1,2]. Globally, HIS have been used in primary health care facilities to record, analyze, report, and transfer patients’ data, in which the data will be highly important to improve the health system’s management [3]. The World Health Organization (WHO) report in 2010 highlighted the importance of HIS as a contributing factor to overall health management improvement [4]. Studies from low- and middle-income countries show that HIS implementation has been carried out, despite challenges and barriers to successful implementation [5].

Indonesia is the world’s fourth most populous country with over 270 million people residing across more than 17,000 islands [6,7]. The country has adopted a decentralized administrative system since 1999, which granted provincial governors and district leaders the authority to govern their own areas, including health care service governance [8,9]. At the national level, the Ministry of Health plays a vital role in formulating health policies, regulations, and strategies that guide the entire health care system, setting standards, coordinating resources, and implementing national health programs. This responsibility extends to the provincial level, where each of the provinces has its own health office (provincial health office) responsible for implementing region-specific health care policies and programs. Additionally, at the district level, the 514 district health offices (DHOs) play a crucial role in managing and monitoring the provision of health care services [8,9].

The smallest unit of public health care service at the subdistrict level is called Puskesmas (Pusat Kesehatan Masyarakat or public health center), which stands for the primary health care service for the local community. The number of Puskesmas increased by 9.3% over the past decade, from 9510 in 2012 to 10,260 in 2021 [10,11]. The Puskesmas are spread across all areas of Indonesia, with the ratio of Puskesmas per subdistrict currently at 1.04, which means that in each subdistrict, there is at least one Puskesmas [7]. Puskesmas serve as the first point of contact for individuals seeking health care, providing preventive, curative, and rehabilitative services in the area of general medicine, maternal and child health, family planning, immunization, and more [12,13]. At the same time, each Puskesmas has the obligation to record and report all health program activities or health data within its working area, using digital HIS or manual reports, and submit it regularly to the DHO [14].

It is crucial for Puskesmas to ensure that robust HIS can accommodate the dynamic monitoring of health problems and interventions, supporting the provision of effective primary health care services for the community. HIS facilitate the collection, storage, analysis, and retrieval of health-related data, empowering Puskesmas and the DHO in making informed decisions within their working area [15]. Thus, Puskesmas can better manage patient records, track disease outbreaks, plan resource allocation, and contribute to the overall improvement of health care services in Indonesia [16,17]. Maintaining a timely and accurate data collection infrastructure, policy makers can then better allocate resources, plan health care services, and implement targeted interventions that address the health care challenges faced by the growing population [18-20].

To date, the use of HIS continues to grow across Indonesia, including in Puskesmas. However, only limited publications are available that discuss the national-scale HIS implementation in Indonesia. A study reported on the implementation of 12 selected HIS in Puskesmas at the national level [21]. Most studies discussed the HIS utilization only for specific programs, such as tuberculosis [22], immunization [23], malaria [24], and electronic medical record system [25,26]. Many studies only focus on HIS implementation in specific areas such as provinces [27,28]. A literature review highlights the utility of HIS but is limited to the Puskesmas scale and only focuses on Puskesmas’ management information systems [27,29]. A narrative study in 2015 reveals the barriers and challenges of HIS implementation in a low- and middle-income country (LMIC) on a general basis [3].

This paper aims to provide, for the first time, a comprehensive national overview of the utilization of HIS across Puskesmas in Indonesia. It presents a descriptive mapping of the types and number of HIS in use, the institutional and infrastructural conditions that may be associated with their implementation, and the common challenges encountered at the facility level. The analysis includes aspects such as internet availability, accreditation status of Puskesmas, system usability, and the availability of training. Additionally, the study explores key workforce characteristics, including staff education background and reported HIS-related workloads. While the study does not assess system interoperability, causality, or performance impact, it offers a foundational evidence base to inform future digital health strategies and more targeted research.

## Methods

### Data Collection

A cross-sectional approach was chosen, using a survey carried out between January 28 and February 28, 2022. The population study covered 10,260 Puskesmas across 34 provinces in Indonesia. Slovin calculator [30] was used to count the sample size based on 5% margin of error. The Slovin formula was used in this study as it is widely used in public health and social science research, where the total population size is known but the population’s variance or SD information is limited. The Slovin formula offers a practical and transparent means of estimating a representative sample size. Although it does not explicitly account for CI or statistical power, this limitation was addressed by selecting a conservative margin of error of 5%, in line with the accepted research standard. Accordingly, an expected sample was 385 participants out of 10,260 Puskesmas. To account for potential data incompleteness, an additional 10% was added, resulting in a final minimum sample size of 424 Puskesmas.

A questionnaire was used to obtain the data from the participants. The questionnaire was developed referring to the PRISM (Performance of Routine Information Systems Management) framework [31] to assess routine HIS in LMICs. We used and adopted the routine health information tools overview tool to list the information systems that exist in the country and the type of data they collect. A pilot survey test was conducted to ensure questionnaire validity and reliability with a total of 20 participants, resulting in a satisfactory Cronbach α value (0·76). The questionnaire consisted of two sections: (1) a list of HIS used by Puskesmas, which developed the HIS, and the utilization and challenges during HIS implementation; and (2) an exploration of factors that influence the utilization of HIS in Puskesmas, including human resources, supporting facilities, and infrastructure. The identities of participants were also captured as representatives from Puskesmas. As the survey relied on self-reporting from designated Puskesmas personnel, responses may reflect individual perceptions or institutional biases rather than objective system evaluations. To reduce response bias, the questionnaire was anonymized, and standardized definitions were provided throughout.

### Data Analysis

Univariate analysis was conducted using SPSS (IBM Corp) software to examine the HIS utilization in Puskesmas across Indonesia. The major islands in Indonesia were divided into six categories: (1) Java Island, covering provinces such as Banten, DKI Jakarta (special capital region of Jakarta), West Java, Central Java, Yogyakarta Special Region, and East Java; (2) Bali and Nusa Tenggara, including Bali, East Nusa Tenggara, and West Nusa Tenggara provinces; (3) Sumatera, covering Aceh, North Sumatra, West Sumatra, Riau, Riau Islands, Jambi, South Sumatra, Bengkulu, Bangka Belitung, and Lampung provinces; (4) Kalimantan, including West Kalimantan, Central Kalimantan, South Kalimantan, East Kalimantan, and North Kalimantan provinces; (5) Sulawesi, including South Sulawesi, North Sulawesi, Central Sulawesi, Gorontalo, Southeast Sulawesi, and West Sulawesi; and (6) Maluku and Papua, consisting of Maluku, North Maluku, Papua, and West Papua provinces.

Puskesmas are systematically distributed across various regions, ensuring accessible health care services are available to all communities. Based on Article 21 of the Ministry of Health Decree Number 75 Year 2014 [12], Puskesmas are divided into three characteristic regions, which are urban, rural, and remote areas. To explore factors associated with HIS implementation in Indonesia, we used the average number of HIS [30] to calculate the degree of association of some risk factors. As the study uses a cross-sectional design, it captures association at a single point in time and cannot establish causality between factors such as training frequency, accreditation, or geographic location and HIS use.

Box plots were used as data visualization tools to depict the average number of HIS used in Puskesmas, along with the minimum and maximum numbers across 34 provinces. Cross-tab analysis was used to examine the frequency of HIS utilization based on the type of Puskesmas’ geographic characteristics (urban, rural, and remote) and accreditation level. Puskesmas accreditation level in Indonesia is categorized as follows: (1) not accredited, (2) basic accreditation (dasar), (3) medium accreditation (madya), (4) major accreditation (utama), and (5) comprehensive accreditation (paripurna).

### Ethical Considerations

We declare that the data collected for this paper do not require ethical approval as no individual data are presented and informed consents were collected during the survey implementation. As such, this research qualifies for exemption from full ethical review in accordance with the provisions outlined in the 2021 Pedoman dan Standar Etik Penelitian dan Pengembangan Kesehatan Nasional (2021 National Health Research and Development Ethical Guidelines and Standards), issued by the Komite Etik Penelitian dan Pengembangan Kesehatan Nasional (KEPPKN)—National Health Research and Development Ethics Committee—under the Indonesian Ministry of Health [32]. Specifically, the exemption applies to studies that involve no personal identifiers, pose minimal to no risk, and focus on evaluating or monitoring public programs, as outlined in the guideline’s section on research exemption criteria. These provisions are consistent with the earlier 2017 guideline and remain the current ethical standard [33]. In accordance with these criteria, all survey responses were anonymized upon collection, and no individual or facility-level identifiers were retained in the analytical dataset. Informed consent was embedded at the beginning of the survey, which provided thorough information about the study and what to expect from the participants. It also states that participation was entirely voluntary and no compensation was given in any form. Data were securely stored on encrypted servers with restricted access, ensuring the confidentiality and integrity of the dataset. The exemption status of this study was reviewed and confirmed by the Digital Transformation Office of the Indonesian Ministry of Health.

## Results

### Number of HIS Used in Puskesmas

A total of 2616 public health centers (25.5% of the total 10,260 public health centers in Indonesia) spread across 320 districts or cities from 34 provinces participated in the survey. Provinces with the highest number of participating health centers were Central Java (326), Lampung (227), and Aceh (214). Among the health centers, 779 of 2700 (28.85%) were located in urban areas; 1143 of 2700 (42.33%) in rural areas; and 475 of 2700 (17.59%) in remote areas.

**Figure 1.**
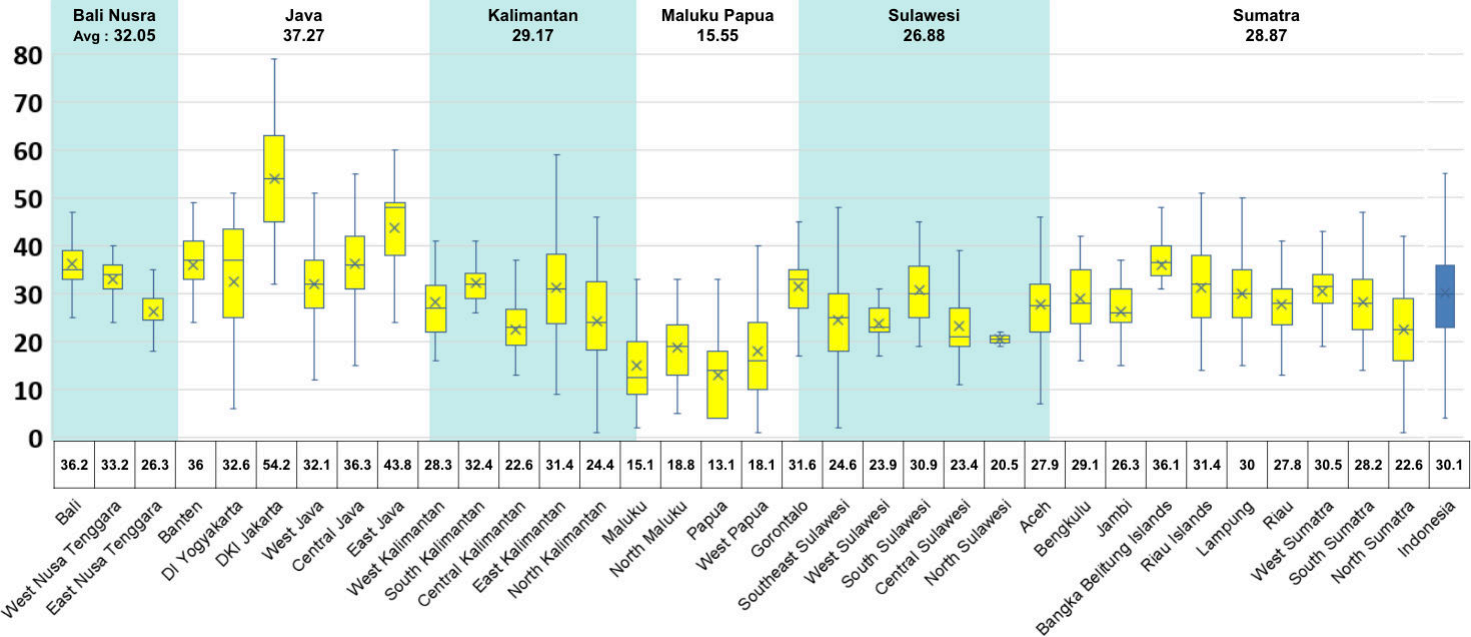
Average number of health information systems used by public health centers based on provinces and major islands.

Based on the study results, the average number of HIS used in public health centers nationwide was 30 (SD 11.2). However, this number varied across provinces. The province with the highest average number of HIS was DKI Jakarta, with 54 (SD 12.6) systems deployed and used for recording and reporting various health services, followed by East Java (43, SD 8.0), Central Java (SD 9.7), Bali (SD 8.7), Bangka Belitung Islands (SD 6.5), and Banten (SD 7.6), which were all around 36 systems in average, while Papua had the lowest average of 13 (SD 9.1) systems. The variation of the number of HIS used by Puskesmas can also be observed based on the distribution of major islands, with Java having the highest average of 37 (SD 11.3) systems, followed by Bali Nusra (32, SD 7.8), Kalimantan (29, SD 10.3), Sumatra (28, SD 8.8), Sulawesi (26, SD 8.5), and the lowest being 15 (SD 9.5) in Maluku Papua (Figure 1).

On average, 72.94% (n=62,060) of the HIS used were provided or developed by the Indonesian Ministries (National Government), making the central government the primary contributor to the abundant number of different HIS available. City or district governments also play a significant role, contributing to 17.45% (n=14,844) of the HIS development, while the provincial government accounts for 5.27% (n=4485). Moreover, third-party organizations and other stakeholders contributed 4.33% (n=3685) and Puskesmas 0.01% (n=12) to the overall HIS deployment. A total of 75.07% (n=63,872) of the HIS were from health-related government offices, while 24.93% (n=21,214) were from non–health-related (eg, the Ministry of Internal Affairs, Ministry of Communication and Informatics, Ministry of Finance, Local and Provincial Governments).

Based on the study, the HIS used by public health centers was 91.54%, which aligned with their needs, and 90% of the data collected has been used as a reference for decision-making, health policy formulation, or program development. However, there exist challenges in using the existing HIS as depicted in the following graph.

**Figure 2.**
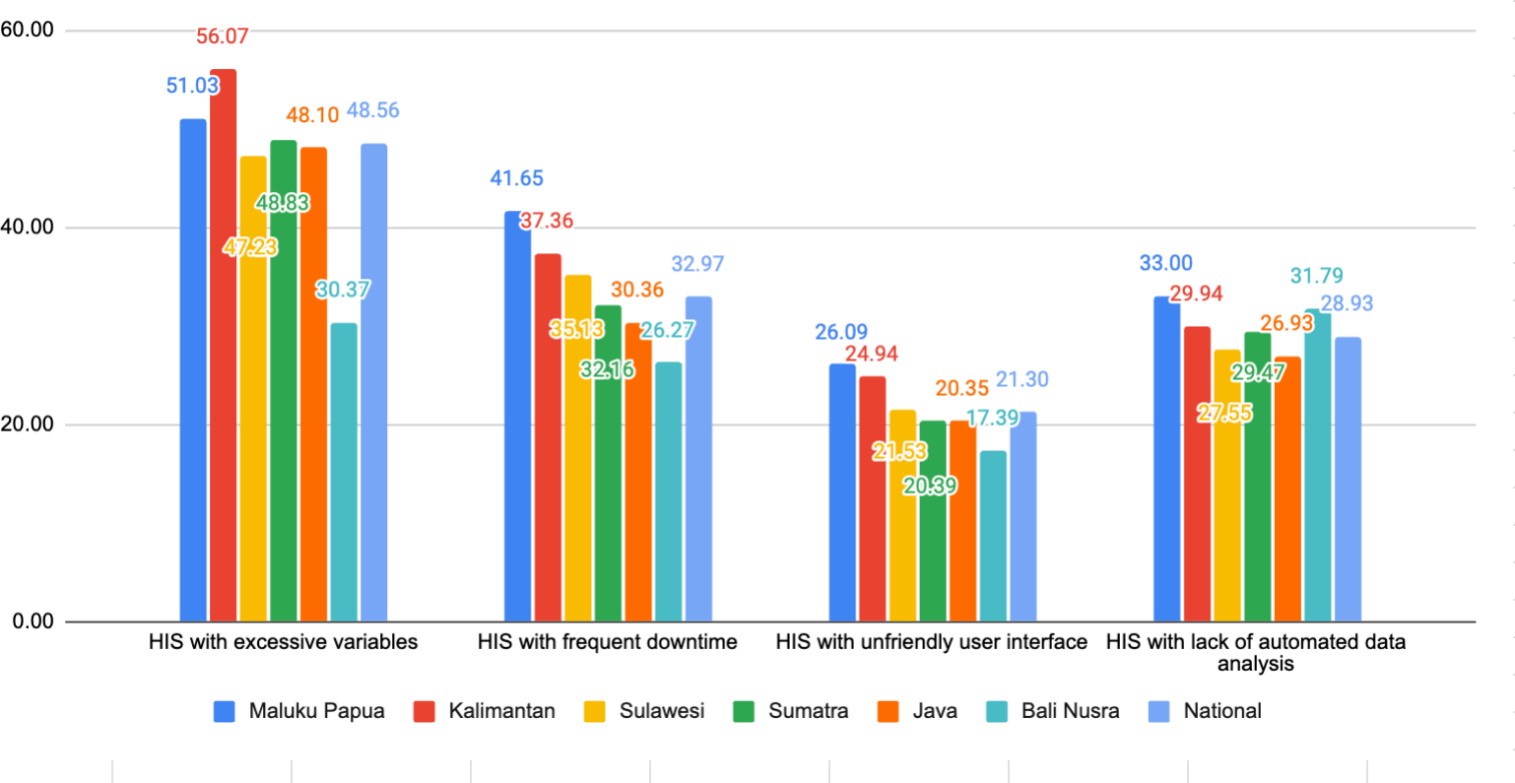
Several limitations of existing HIS based on major islands. HIS: health information systems.

The findings depicted in Figure 2 revealed major challenges of the existing HIS used by Puskesmas across Indonesia. Approximately, 49% (n=132,300) of all HIS in the country had excessive data input variables, leading to prolonged form-filling, extended patient registration queue times, and increased screen time for health care personnel. In addition, 33% (n=89,100) of the systems experience frequent downtime, posing challenges in health care service delivery and data management, 21% (n=56,700) of HIS were not user-friendly, and 29% (n=78,300) of HIS did not have the ability to generate automated data analysis.

**Figure 3.**
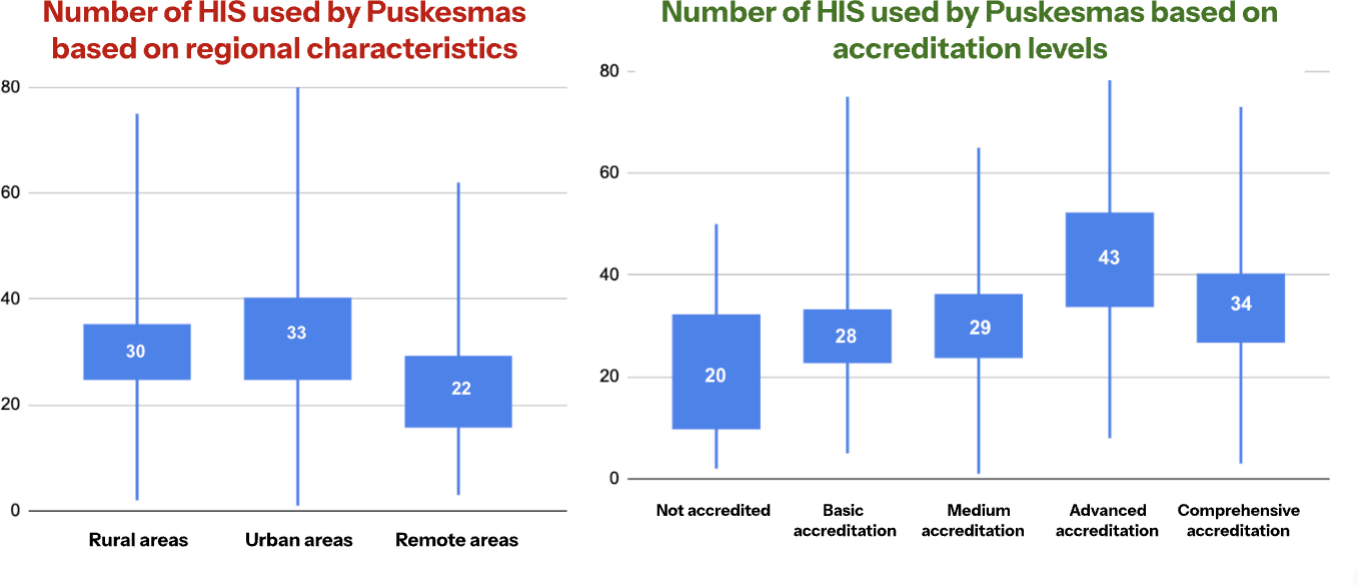
Number of HIS used by Puskesmas based on geographic characteristics and accreditation levels. HIS: health information systems.

Public health centers in rural areas used an average of 30 different HIS, while urban areas used 33 systems, and remote areas used 22 systems (Figure 3). Furthermore, based on the accreditation level, public health centers with no accreditation used an average of 20 different HIS, while Puskesmas with basic (*dasar*), medium (*madya*), accreditation (*utama*), and comprehensive accreditation (*paripurna*) levels used 28, 29, 43, and 34 systems, respectively.

### Factors Influencing HIS Utilization Across Public Health Centers

#### Internet Availability

**Figure 4.**
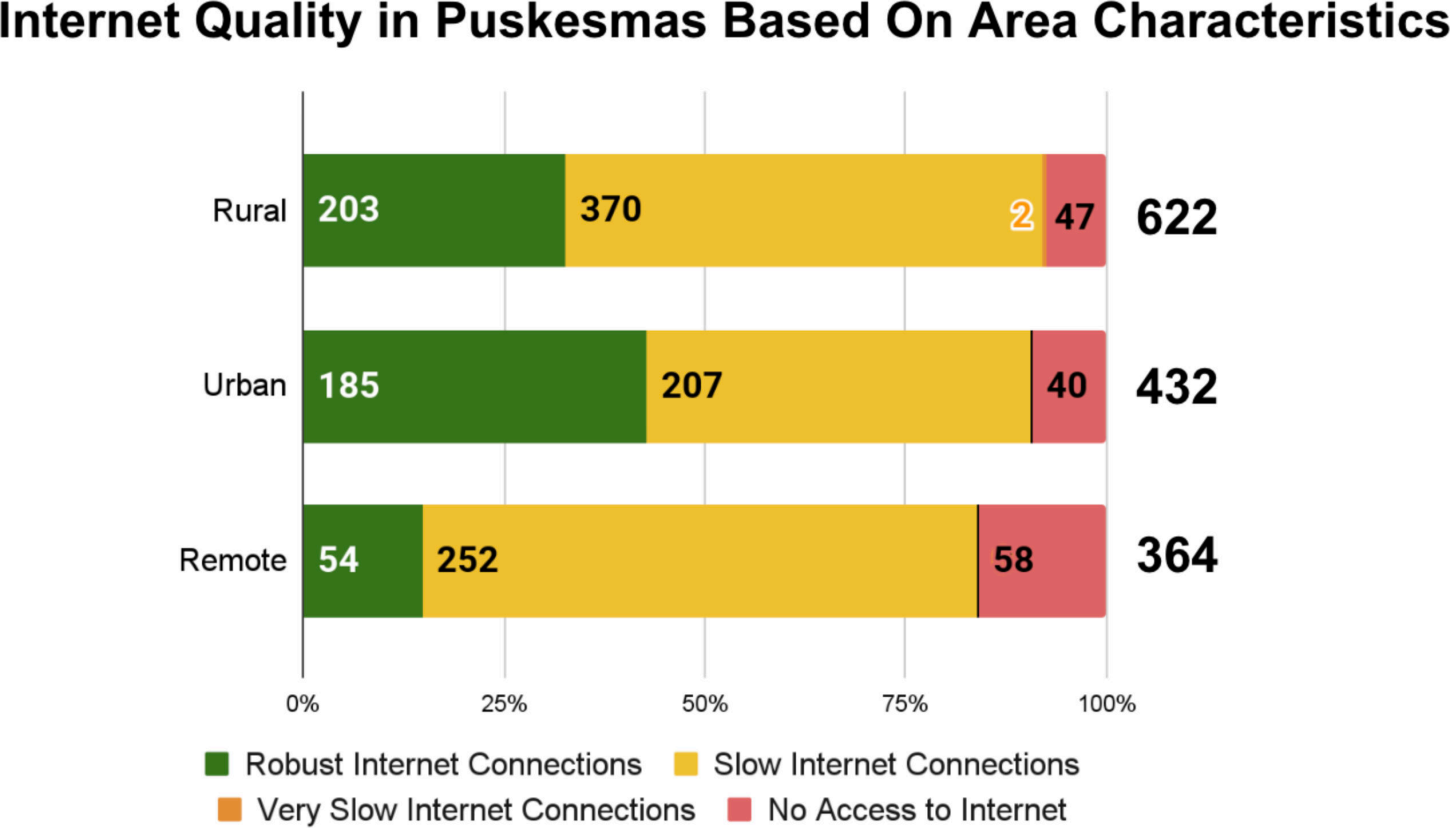
(A) Internet quality in public health centers and (B) types of internet facilities used by health care personnel to access HIS in Puskesmas. (C) Internet quality in public health centers based on area characteristics. HIS: health information systems.

A separate analysis was carried out to explore factors influencing HIS utilization. Based on the survey, 9.45% (138/1460) of Puskesmas had no access to the internet. Specifically, the majority of Puskesmas have internet access, but only 28.9% (422/1460) have access to robust and efficient internet connections; meanwhile, 51.1% (746/1460) reported slow internet connections, and 10.55% (154/1460) reported very slow internet connections (Figure 4A).

Figure 4B shows the types of internet facilities mostly used by health care personnel to access HIS in Puskesmas. Among the surveyed Puskesmas (n=1309), 417 (31.88%) relied solely on Puskesmas Wi-Fi for their internet access to input the health data reports; 280 (21.40%) used Puskesmas Wi-Fi and private home internet; and a large portion reported using a combination of Wi-Fi, private home internet, and internet credit fund paid by Puskesmas (422/1309, 32.27%). Figure 4C depicts that in urban areas, 42.8% (185/432) of Puskesmas have robust internet connections, while 47.9% (207/432) experience slow internet, and only 9.3% (40/432) have no access to the internet. In contrast, in remote areas, only 14.84% (54/364) of Puskesmas have robust connections, whereas 69.2% (252/364) experience slow internet, and 15.93% (58/364) have no access.

#### Human Resources

Human resources are pivotal in affecting the utilization and adoption of HIS. Table 1 depicts the background education of HIS managers in Puskesmas. Out of the total surveyed individuals (n=1330), 344 (25.86%) of HIS managers were from other health care professions such as nutritionists and pharmacists, followed by nurses (327/1330, 24.59%), public health staff (255/1330, 19.17%), non–health care professionals such as informaticians and computer technicians (241/1330, 18.10%), and midwives (147/1330, 11.05%).

**Table 1.** Educational background of HIS^a^ managers in public health centers (n=1330).

Educational background of HIS managers	HIS personnel, n (%)
Other health care personnel	344 (28.86)
Nurse	327 (24.59)
Public health personnel	255 (19.17)
Nonhealth care personnel	241 (18.12)
Midwife	147 (11.05)
Doctor	14 (1.05)
Pharmacist	2 (0.15)

a HIS: health information systems.

Table 2 provides insights into the number of HIS or programs input by Puskesmas personnel. Among the surveyed Puskesmas (n= 850), the data showed that 22.71% (193/850) of Puskesmas required each health personnel to input data into one to two different HIS. Additionally, 188 (22.12%) of the Puskesmas personnel managed and input data into more than six different HIS or programs, while 159 (18.71%) of them handled one to three programs.

**Table 2.** Number of HIS^a^ programs input by public health centers personnel (n=850).

HIS programs input by public health center personnel	Puskesmas, n (%)
More than 6 programs	188 (22.1)
1‐6 programs	79 (9.3)
1‐5 programs	91 (10.7)
1‐4 programs	87 (10.2)
1‐3 programs	159 (18.7)
1‐2 programs	193 (22.7)
1 program	53 (6.2)

a HIS: health information systems.

Further analysis shows that 74.30% (1133/1525) of personnel in Puskesmas only received training at the initial system’s installation, 13.64% (157/1525) indicate that the training only occurs once a year, and 10.30% (157/1525) report that there was no training available for data input and management. Furthermore, 80.51% (1225/1521) of respondents report the existence of a knowledge transfer process, highlighting the significance of sharing expertise and information among the staff.

Table 3 presents a bivariate analysis of various characteristics influencing the adoption of information systems in public health centers. We categorized the below and above average number of HIS (30 HIS as a cutoff) and conducted binary logistic regression analysis on SPSS. Key variables include geographic factors (main islands and geographic characteristics), accreditation levels, training frequency, knowledge transfer, number of HIS programs input by personnel, and internet quality. Among geographic factors, health centers in Java show the highest likelihood of having >30 information systems (odds ratio [OR] 32.85, 95% CI 14.9-72·41) compared to Maluku Papua. Urban areas have a greater likelihood of higher system adoption compared to rural and remote areas (OR 4.73, 95% CI 3.43-6·50). Accreditation levels are strongly associated with system adoption, with comprehensively accredited centers being the most likely to use >30 systems (OR 10.20, 95% CI 3.41-30.51). Training frequency also plays a role, where biannual training is significantly associated with higher adoption (OR 1.77, 95% CI 1.01-3.08). Knowledge transfer presence slightly increases the likelihood of greater system use (OR 1.33, 95% CI 0.97-1.81). The number of HIS programs input by personnel does not show a strong pattern. Internet quality does not exhibit a substantial impact, as OR values remain close to 1.

**Table 3.** Factors influencing HIS^a^ utilization.

Characteristics	Total Puskesmas (n)	Below average (≤30 HIS), n (%)	Above average (>30 HIS), n (%)	Univariable, OR^b^ (95% CI)
Main island				
Maluku Papua	175	162 (92.6)	13 (7.4)	1 (reference)
Kalimantan	231	163 (70.6)	68 (29.4)	6.18 (2.82‐13.52)
Bali Nusra	315	187 (59.4)	128 (40.6)	5.53 (2.05‐14.89)
Sulawesi	956	567 (59.3)	389 (40.7)	3.54 (1.56‐7.98)
Sumatra	106	33 (31.1)	73 (68.9)	6.25 (2.94‐13.28)
Java	645	149 (23.1)	496 (76.9)	32.85 (14.9‐72.41)
Geographic characteristics				
Remote areas	595	458 (77)	137 (23)	1 (reference)
Rural areas	1077	505 (46.9)	572 (53.1)	3.41 (2.54‐4.59)
Urban areas	748	294 (39.3)	454 (60.7)	4.73 (3.43‐6.50)
Accreditation levels				
Not accredited	154	114 (74)	40 (26)	1 (reference)
Basic accreditation	513	291 (56.7)	222 (43.3)	2.26 (1.22‐4.21)
Medium accreditation	1273	695 (54.6)	578 (45.4)	1.54 (0.86‐2.7)
Advanced accreditation	400	146 (36.5)	254 (63.5)	2.82 (1.47‐5.41)
Comprehensive accreditation	88	15 (17)	73 (83)	10.2 (3.41‐30.51)
Training frequency				
No training	158	105 (66.5)	53 (33.5)	1 (reference)
Occurs at the beginning of system socialization	3	2 (66.7)	1 (33.3)	0.24 (0.01‐3.23)
Once a year	1067	528 (49.5)	539 (50.5)	1.4 (0.86‐2.27)
Twice a year	209	114 (54.5)	95 (45.5)	1.77 (1.01‐3.08)
Knowledge transfer				
No knowledge transfer process	282	178 (63.1)	104 (36.9)	1 (reference)
Knowledge transfer processexistst	1140	558 (48.9)	582 (51.1)	1.33 (0.97‐1.81)
Number of HIS or programs input by public health center personnel				
1	61	34 (55.7)	27 (44.3)	1 (reference)
1‐2	197	111 (56.3)	86 (43.7)	1.38 (0.74‐2.54)
1‐3	161	72 (44.7)	89 (55.3)	0.82 (0.57‐1.2)
1‐4	91	44 (48.4)	47 (51.6)	1.1 (0.72‐1.66)
1‐5	94	49 (52.1)	45 (47.9)	1.08 (0.64‐1.81)
1‐6	77	43 (55.8)	34 (44.2)	0.72 (0.41‐1.25)
≥6	670	336 (50.1)	334 (49.9)	1.06 (0.58‐1.93)
Internet quality				
No access to internet	121	63 (52.1)	58 (47.9)	1 (reference)
Very slow internet connections	120	54 (45)	66 (55)	1.27 (0.74‐2.16)
Slow internet connections	711	362 (50.9)	349 (49.1)	1.04 (0.69‐1.56)
Robust internet connections	367	194 (52.9)	173 (47.1)	1.03 (0.67‐1.59)

a HIS: health information systems.

b OR: odds ratio.

## Discussion

### Principal Findings

HIS are foundational to modern health care delivery and serve as critical tools for supporting evidence-based decision-making and policy development [34]. Well-functioning HIS should not only collect health data but also enable the analysis, synthesis, and timely dissemination of information across all components of the health system [35]. Previous studies have demonstrated that effective HIS use can contribute to improved care coordination, more efficient facility management, and enhanced patient safety outcomes [34-36]. While most existing research has focused on hospital-level or disease-specific HIS implementations, this study offers the first national-level descriptive mapping of HIS utilization across Indonesia’s public primary health care centers (Puskesmas). It identifies the number and types of systems in use and highlights contextual factors that may influence HIS adoption and implementation at the facility level.

Various innovations in resource-limited areas are also making significant investments in national HIS. These initiatives are anticipated to enhance access to quality health care and contribute to the reduction of overall costs in the health care sector) [37-41]. This study reveals that there was a massive, recent development of multiple HIS or digital apps within Indonesia, driven by government initiatives (at all levels of government, from central, provincial, to district). In particular, massive development of HIS in Indonesia is caused by different needs of a key performance indicator for each division or directorate of the central government in Indonesia. Thus, there have been increased numbers of projects to develop different digital apps and HIS that focus on specific diseases, such as SITB (Sistem Informasi Tuberkulosis), for tuberculosis information system; SIHA (Sistem Informasi HIV-AIDS), for HIV-AIDS information system; SIHEPI (Sistem Informasi Hepatitis), or Hepatitis Information System; SIPTM (Sistem Informasi Penyakit Tidak Menular), or NonCommunicable Disease Information SysteM; SIPD3I (Sistem Informasi Penyakit yang Dapat Dicegah dan Diobati dengan Imunisasi), or Vaccine Preventable Diseases Information System; ESISMAL (Electronic System of Malaria), or Malaria Information System.

Digital app development initiatives were also increased due to a new specific program introduced by the central government to monitor specific programs, such as PISPK (Program Indonesia Sehat Pendekatan Keluarga), or the Healthy Family Program; SISTBM (Sistem Informasi Sanitasi Total Berbasis Masyarakat), or Health Sanitation Population-Based; e-PPGBM (Electronic Pencatatan dan Pelaporan Gizi Berbasis Masyarakat), or Nutrition Information System; SKDR (Sistem Kewaspadaan Dini dan Respon), or Early Warning of Epidemic Diseases; ASPAK (Aplikasi Sarana Prasarana dan Alat Kesehatan), or Health Facilities and Supplies Application; P-Care (Primary care), a medical record for health insurance; HFIS (Health Financing Information System), for financing of health insurance, and many more introduced by each provincial or district level due to decentralization health system regulation [21,37]. This greatly magnifies the fragmentation, disparities, burden for health care workers, fragmented data, lack of data integration and interoperability, and low level of system standardization [37-40]. Another common HIS in Puskesmas is also called SIMPUS (Sistem Informasi Manajemen Puskesmas), or Puskesmas Management Information System, which manages both patient electronic medical record management and administrative matters ranging from human resources, finance and planning, pharmaceuticals and medical devices, and reporting [42].

Based on Articles 3 and 6 of the Ministry of Health Decree Number 31 Year 2019 [15], SIMPUS is part of the district information system. In order to simplify all the different existing apps or information systems used by health care facilities, including Puskesmas, a national HIS (Sistem Informasi Kesehatan Nasional) is currently being developed by the Ministry of Health of Indonesia [43]. The systemic goal is to allow the deployment of Fast Healthcare Interoperability Resources-based standardization to be used by health care facilities’ HIS developers to connect all health care institutions’ services at all levels in the whole country [43]. In the context of primary health care, each Puskesmas and DHO has the opportunity to customize and enhance its SIMPUS framework as long as it complies with the Sistem Informasi Kesehatan Nasional standard, to better address its unique priorities, ensuring that the system effectively meets the diverse needs of its community. This initiative enables each district to confidently engage a diverse array of information technology developers.

The large number of HIS used is a major challenge. At the simplest level, the amount of different HIS affects the responsibility burden of each health worker, from data recording to reporting [44]. The reporting obligation of Puskesmas is centrally mandated; however, using different numbers of HIS adds to the medical staff workload. The study shows an average number of 30 different HIS used for various health data reporting functions, indicating a high burden for health care personnel to input data into different systems that are not integrated. Inevitably, this landscape of multiple siloed systems leads to duplication in data entry. It impacts the allocation of adequate time for health workers to deliver health care services due to data recording and reporting tasks [45].

In addition, the results suggest different numbers of HIS used for data reporting across provinces and major islands in Indonesia. This would create a technical challenge to future data integration attempts at a central government level. Java and Sumatra Island tend to use more HIS compared to Bali, Nusra, and other islands. This reflects the relative population distribution (much higher population density in Java and Sumatra in the western region of Indonesia) and is thus influenced by several factors such as the availability of human resources, infrastructure, organizational support, aid funding, and program priorities [45-47].

Considering the experiences of other countries, it seems that one of the root causes of the failure of HIS is the absence of adequate information and communication technology infrastructure [48]. A previous study shows that adequate access to the internet, computer availability, and availability of electricity play roles in the success rate of HIS utilization [49]. Findings in Haiti also show that the important factor for long-term implementation success of complex information systems is balancing investments in hardware and software infrastructure upkeep [50], and many other studies suggested that HIS utilization will be optimal, effective, and efficient when they are supported by adequate facilities and infrastructure [46-49]. In spite of the potential and opportunities that lie in HIS to transform the health care sector, including helping to foster the development of evidence-based policy, many challenges are evident and imminent. These challenges range from issues related to the technology infrastructure, human resources, system users, and financial constraints [49,51].

Based on the study results, health care workers felt that the variables of data that need to be recorded and reported in the HIS are too many, with complicated user interfaces. Data input with long required variables for reporting caused data duplication and poor usability of HIS [45,52]. Poor usability has been linked to a reduced acceptance of digital systems in the long term, increased errors, and reduced user efficiency and can even adversely affect patient safety [52,53]. The International Standard Organization has defined the usability of systems with three features of effectiveness, efficiency, and satisfaction, and user interface problems can be associated with all of these features [54]. Previous studies have shown that usability problems, such as overcrowded pages and many steps to perform a task, entail reduced user productivity [55,56]. Another study in Iran showed that HIS “flexibility and efficiency of use” and “consistency and standard” are problematic [3,5]. Furthermore, diversity in the presentation and visualization of data variables can result in ineffective communication and data reporting. For Indonesia, where universal health care coverage was introduced only recently in 2014, it is critical to use common standards and observe the consistency of data variables across the whole system [57]. Moreover, presented information in any format should be self-explanatory, so that the users can initiate actions at any time without recalling their experience from the previous use of the system [58].

Health care organizations are expected to increasingly depend on business intelligence tools, including “dashboards,” to capture, analyze, and present data on performance metrics [59]. For example, dashboards allow users to quickly visualize actionable data to inform and optimize clinical and organizational performance [60]. However, the multiplicity of HIS, and as such, the multiplicity of produced dashboards, is likely to impede the utilization of collected data. Thus, the data management problem is one of the important findings in this study, especially the lack of automated data analysis generation through the dashboard of the existing HIS. In reality, dashboards are typically embedded in complex health care organizations with massive data streams, servicing end users with distinct needs. Thus, designing effective dashboards is a challenging task, and the theoretical underpinnings of health care dashboards are poorly characterized; even the concept of the dashboard remains ill-defined [55]. Several LMICs face the same problem of insufficient quality of data produced by the systems that limit their usefulness with regard to decision-making [61-63].

The continuity of using HIS during routine operations depends on the information technology infrastructure. The discrepancies in communication technology infrastructure availability across the 34 provinces in Indonesia remain one of the obstacles when it comes to the need for a stable internet connection, even in urban areas that already have communication infrastructure with 5G networks [64]. The limitations of the internet coverage and limited network capacity impact HIS operability, since it may limit the network coverage area to only the registration counter [49]. Frequent downtime of HIS was found in this study, showing the need for further improvements in infrastructure and facilities. This lack of and instability of information technology infrastructure, such as the limitation of internet access, electricity supply, and availability of computers, was similar to that found previously in Brazil, Sub-Saharan Africa, and Tanzania [65-67].

Finally, human resources play a pivotal role in implementing the HIS in Puskesmas. However, the IT personnel remain one of the challenges to date, as the procurement of IT personnel in a primary health care center is not a mandatory requirement yet in Indonesia. This absence of highly trained IT personnel could be mitigated by well-trained health care staff; however, this is not the case, as most staff have reported inadequate amounts of training. According to this study, the amount of HIS managed by each staff ranges from one to six, combining the possibility of suboptimal handling of HIS with existing high workloads [49]. This is similar to studies in Saudi Arabia, Malaysia, and other LMICs [68,69].

Further analysis identified factors associated with the utilization of HIS. However, due to its cross-sectional nature, the study does not allow us to determine the direction or causality of observed relationships between institutional characteristics and HIS adoption. For example, while higher accreditation levels or urban status are associated with higher HIS use, these findings should be interpreted as correlational, not causal. Table 3 indicates that Java Island has a significantly higher number of HIS utilization (>30 information systems) compared to Sumatera, Kalimantan, Sulawesi (central part), and Maluku and Papua (eastern part). This pattern is common in LMICs where the numbers of HIS utilization are strongly influenced by several factors such as human resources, institutional funding, foreign aid, transparency, and prioritization issues [49]. Table 3 also shows the disparities in the numbers and percentages of HIS utilization based on geographical characteristics. In general, urban and rural areas had more HIS utilization than remote areas. The existence of information technology infrastructure remains the main challenge in LMICs, especially in rural and remote areas. These areas often have limited or no internet access, computer availability, and electricity supply [49]. The lack of stable and affordable internet connectivity can be an obstacle in using the HIS for sending and receiving health data effectively [33]. The findings indicate that Puskesmas with slower internet connectivity are making considerable efforts to effectively use the existing HIS. Interestingly, their performance appears slightly better than that of Puskesmas with more robust internet connectivity. However, it should be noted that the majority of data input in areas with limited internet access is primarily conducted by the DHO.

This study has also evaluated the effectiveness of health care organization accreditation [38]. A previous study shows that accreditation leads to improved quality of care, better strategic planning, enhanced human resource management, stronger leadership, improved archiving, and increased patient satisfaction [38]. Based on our findings, the top level of accreditation status uses more numbers of HIS than the other levels of accreditation (medium and basic).

The human resource and capacity factor plays a vital role in the effectiveness of information systems implementation. This study demonstrates that when Puskesmas provides regular training and facilitates knowledge transfer among staff regarding the HIS, there is a marked increase in the system’s utilization. This emphasizes the significance of ongoing education and support in optimizing the advantages of information systems. Like other studies conducted in Ethiopia [38,39], the study findings indicate that the odds of using HIS were significantly higher among primary health care that engaged in regular training and knowledge transfer. This might be due to the fact that health professionals who were trained on HIS had the potential to compile, analyze, and use information generated in the routine day-to-day activities.

Out of the variables that showed no significant difference between the numbers of HIS program input by the Puskesmas staff (1‐5 staff and ≥6 staff) and the numbers of HIS utilization. This might be due to several factors, such as staff awareness, commitment, and motivation [50]. A study conducted in India underlined that, although the utilization of HIS is contingent upon data analysis skills, organizational factors play a crucial role in the effective application of these skills [40]. Reports also showed that strengthening HIS, focusing not only on technical but also behavioral and organizational structures, is one essential component for improving the quality and use of data for decision-making at all levels of the health system [68].

### Strengths and Limitations

The research exhibits several strengths that contribute to its significance in the field. First, it represents the first-ever study of its kind, involving public health centers on a national scale to portray the utilization of HIS. This wide-scale view provides a comprehensive understanding of the current state of HIS implementation in Puskesmas throughout Indonesia. Additionally, beyond the mapping of HIS use, the research highlighted the key challenges faced by field personnel in effectively using HIS, offering insights into potential areas for improvement.

However, certain limitations should be acknowledged. First, while the overall sample size exceeded the national threshold, some provinces (eg, South Sulawesi, East Nusa Tenggara, and Jambi) were significantly underrepresented, limiting generalizability at the provincial level. Second, the cross-sectional nature of the study restricts causal interpretation; relationships observed between institutional characteristics and HIS use should be treated as associative rather than explanatory. Third, as the study relied on self-reported data, it is subject to recall and reporting bias, even though measures were taken to ensure anonymity and standardization. Fourth, the study did not capture qualitative insights from HIS users, which could have provided valuable context around user behavior, system workarounds, and local adaptations. Fifth, while fragmentation of systems was reported, no technical assessment of interoperability, data exchange mechanisms, or governance structures was conducted. Sixth, while basic human resource data were collected (eg, education, training, and HIS load), we were unable to assess the impact of digital workload on staff well-being, task performance, or gender-specific roles. Seventh, although 90% of respondents reported that HIS data supported decision-making, we did not explore how the data were used, by whom, or with what outcomes. Finally, we did not assess the cost-effectiveness or financial sustainability of managing multiple HIS platforms—an area that is especially important for integration planning and national budgeting.

To address these gaps, we recommend that future studies adopt stratified or purposive sampling strategies to ensure adequate representation of undersampled provinces. Longitudinal and mixed method research will be critical to explore causal mechanisms and contextual dynamics of HIS adoption and adaptation. System audits and process evaluations can help validate reported use and assess HIS quality, reliability, and integration performance. Additionally, qualitative interviews with HIS users and implementers—particularly in remote and low-resource settings—can shed light on the lived realities of system implementation and usability. Research on human resource burden, digital competency gaps, and gendered work distribution is also needed to inform equitable capacity building. Finally, economic evaluations and costing studies should be embedded into HIS consolidation efforts to assess long-term sustainability and ensure efficient resource allocation.

Despite these limitations, this baseline study provides critical insights into the HIS landscape in Indonesia and serves as a foundation for evidence-informed policy-making and more effective digital health planning.

### Conclusions

HIS play a significant role in supporting policy-making at public health centers, districts or cities, provinces, and the national level. This study, the first of its kind to assess HIS utilization across more than 2600 Puskesmas nationwide, reveals a highly fragmented digital environment, with health centers operating an average of 30 different HIS platforms—many of which are redundant, nonintegrated, and burdensome to manage. The findings highlight challenges in internet connectivity, system usability, workforce digital readiness, and alignment with service delivery needs. Further analysis in this study suggests that geographic location, accreditation level, and training frequency are among the most influential factors in the adoption of information systems.

Policy interventions must consider the diverse operating environments of Puskesmas to avoid one-size-fits-all approaches. Urban Puskesmas, often with stronger infrastructure and digital capacity, are well-positioned to adopt interoperable dashboards, advanced analytics tools, and integrated HIS platforms aligned with national systems like SATUSEHAT. These centers may also benefit from certification-based training in data analytics and the recruitment of dedicated digital health personnel to enhance system management and interoperability. In contrast, rural and remote Puskesmas operate with limited internet access, power supply, and IT support. For these settings, policy makers should prioritize offline-compatible, simplified HIS that minimizes data entry burdens and operates on low-resource devices, such as mobile phones. Training efforts should focus on basic digital literacy, with peer-to-peer cascading models to enhance local knowledge transfer and sustainability. Infrastructure support, such as solar-powered equipment or network boosters, may also be critical enablers in these areas.

Governance reforms should promote regional coordination, enabling DHOs to provide HIS oversight and technical support, particularly for underserved areas. National initiatives to consolidate and standardize HIS should be complemented with financial and operational feasibility studies to ensure sustainability, especially where overfragmentation currently burdens frontline services.

This baseline study provides a foundational understanding of HIS use and challenges in Indonesia and calls for differentiated, evidence-informed strategies tailored to each health center’s digital maturity and contextual needs. Future research should explore HIS interoperability, real-world data use, user experience, and economic impact to guide Indonesia’s journey toward a more integrated and equitable digital health ecosystem.

## Abbreviations

DHO: district health office
HIS: health information systems
LMIC: low- and middle-income country
OR: odds ratio
PRISM: Performance of Routine Information Systems Management
WHO: World Health Organization
